# Aza-Based Donor-Acceptor Conjugated Polymer Nanoparticles for Near-Infrared Modulated Photothermal Conversion

**DOI:** 10.3389/fchem.2019.00359

**Published:** 2019-05-21

**Authors:** Guobing Zhang, Suxiang Ma, Weiwei Wang, Yao Zhao, Jiufu Ruan, Longxiang Tang, Hongbo Lu, Longzhen Qiu, Yunsheng Ding

**Affiliations:** ^1^National Engineering Laboratory of Special Display Technology, State Key Laboratory of Advanced Display Technology, Academy of Photoelectronic Technology, Hefei University of Technology, Hefei, China; ^2^Key Laboratory of Advanced Functional Materials and Devices of Anhui Province (HFUT), Department of Polymer Science and Engineering, School of Chemistry and Chemical Engineering, Hefei University of Technology, Hefei, China

**Keywords:** conjugated polymer, aza-heterocycle acceptor, near-infrared absorption, nanoparticles, photothermal conversion

## Abstract

It is highly desired that synthesis of photothermal agents with near-infrared (NIR) absorption, excellent photostability, and high photothermal conversion efficiency are essential for potential applications. In this work, three (D-A) conjugated polymers (**PBABDF-BDTT, PBABDF-BT**, and **PBABDF-TVT**) based on aza-heterocycle, bis(2-oxo-7-azaindolin-3-ylidene)benzodifurandione (BABDF) as the strong acceptor, and benzodithiophene-thiophene (BDTT), bithiophene (BT), and thiophene-vinylene-thiophene (TVT) as the donors, were designed and synthesized. The conjugated polymers showed significant absorption in the NIR region and a maximum absorption peak at 808 nm by adjusting the donor and acceptor units. Their photothermal properties were also investigated by using poly(ethylene glycol)-block-poly(hexyl ethylene phosphate) (mPEG-b-PHEP) to stabilize the conjugated polymers. Photoexcited conjugated polymer (**PBABDF-TVT**) nanoparticles underwent non-radiative decay when subjected to single-wavelength NIR light irradiation, leading to an excellent photothermal conversion efficiency of 40.7%. This work indicated the aza-heterocycle BABDF can be a useful building block for constructing D-A conjugated polymer with high conversion efficiency.

## Introduction

Near-infrared (NIR) light has been widely used in sensing, imaging, and biotherapy fields (Qian et al., 2013; Yang et al., 2013; He et al., 2015; Antaris et al., 2016; Song et al., 2016; Zhang et al., 2016), owing to its superior advantages in remote sensing operations, microinvasion, and biological window. Photothermal conversion is the result of the non-radiative transition of excited electrons back to the ground state. (Huschka et al., 2011; Geng et al., 2015). Therefore, photothermal agents with NIR absorption are very important as they can convert NIR light into thermal energy. To date, various photothermal conversion reagents with various NIR absorbance have been explored. Among them, precious metals (such as Au, Ag, and Pt) (Shi et al., 2014; Kim et al., 2015; Marta et al., 2015; Luo et al., 2016), carbon nanomaterials (Yang et al., 2010; Guo et al., 2015), inorganic compounds (Tian et al., 2013), and other materials have shown excellent photothermal properties. However, these materials are not biodegradable and may cause potential long-term toxicity in biological applications (Li et al., 2016a; Zhou et al., 2016). Compared with inorganic materials, organic NIR dyes have attracted considerable attention because of their good biocompatibility and biodegradability (Chen et al., 2013; Zheng et al., 2013). However, severe photobleaching and low photothermal conversion efficiency has hindered their further application. Therefore, organic photothermal agents with a high photothermal conversion efficiency and excellent photostability should be developed. The conjugated polymers showed a sharp and broad absorption peak in the NIR region by adjusting the donor and acceptor units and they have been widely used in organic photovoltaics and field-effect transistors (Günes et al., 2007; Liang and Yu, 2010). As reported, the conjugated polymers displayed excellent photostability, high photothermal conversion efficiency, and good biocompatibility (Pu et al., 2014; Lyu et al., 2017). They also exhibited strong donor–acceptor (D-A) interaction between the nearest neighbor intermolecular overlapping regions and ordered close packing of polymer chains. The D-A structure facilitated strong intramolecular charge transfer (ICT), resulting in efficient fluorescence quenching and high photothermal performance (Guo et al., 2017). Therefore, conjugated polymers are a promising photothermal agent which can effectively utilize the NIR light for photothermal application. For example, Lee and co-workers used the D-A strategy to synthesize a semiconductor nanoparticle with a low bandgap to harvest infrared light. Furthermore, they introduced a porphyrin as a light-harvesting side chain into the backbone, resulting in a record-high photothermal conversion efficiency of 62.3% (Zhang et al., 2017b). Huang and co-workers achieved good planar backbone structure through turning the acceptor unit; it exhibited higher photothermal conversion efficiency of 74% and this is the highest one so far for conjugated polymers (Dong et al., 2018). Within the building block library for conjugated polymer, isoindigo (IIG) is one of the most popular acceptors to construct high-performance semiconductors (Mei et al., 2013; Holliday et al., 2014; Chang et al., 2018). The advantages of IIG as an acceptor unit for D-A conjugated polymers arise from low energy levels, large local dipole, and excellent solubility after N-alkylation (Deng and Zhang, 2014). However, IIG-based conjugated polymers exhibited relatively narrow absorption, the edges of the film absorption bands only extend to about 800 nm. Consequently, the maximum absorption of IID-based polymer showed bad overlap with the NIR window, which has an irradiation at about 808nm. Thus, the exploration of IIG-based conjugated polymers with maximum absorption located in the NIR region is highly desired.

**Figure 1:**
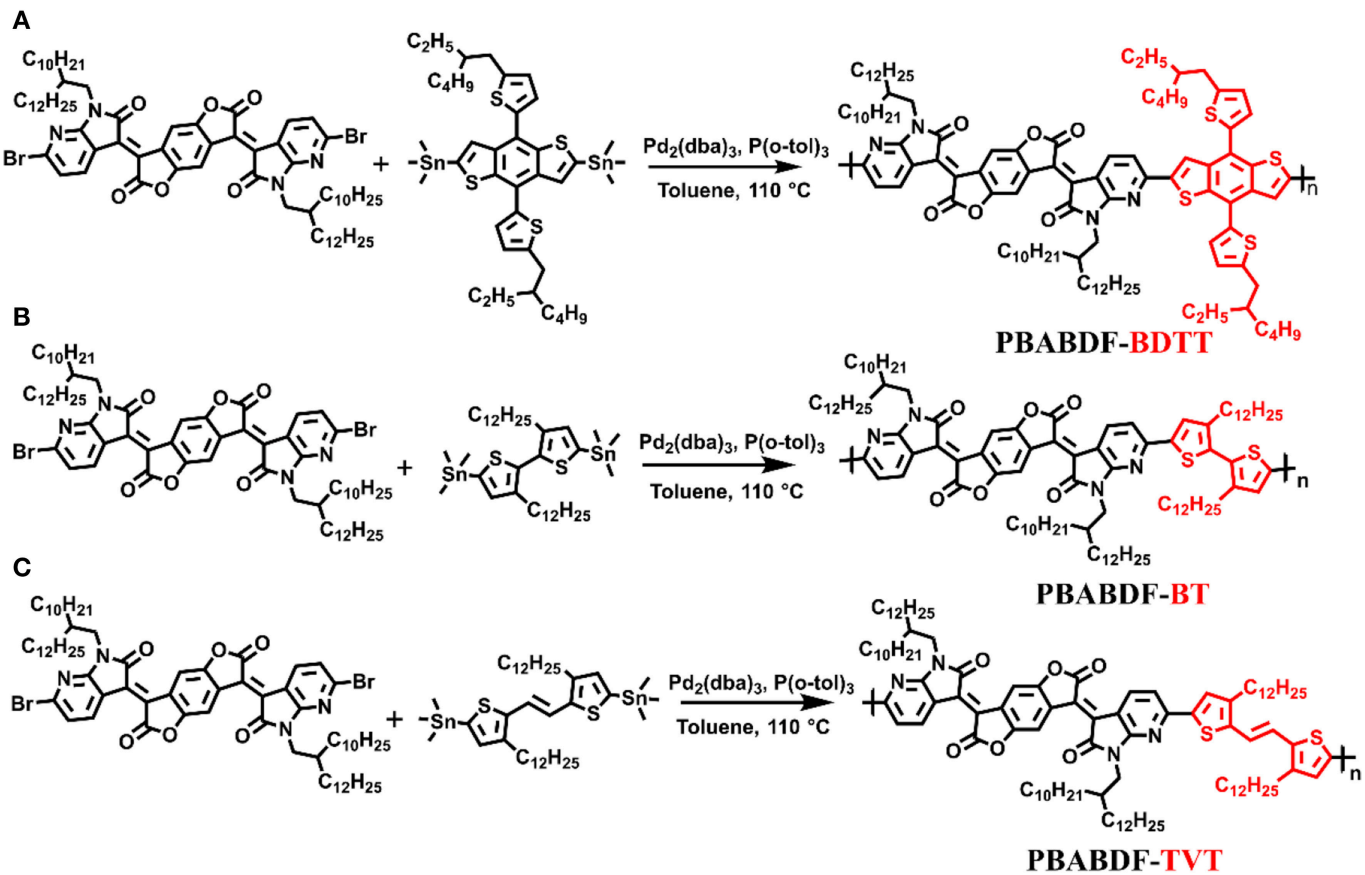
Synthesis pathways for the CPs: PBABDF-BDTT **(A)**, PBABDF-BT **(B)**, and PBABDF-TVT **(C)**.

In our previous work, an IIG derivative unit and its D-A conjugated polymer were designed by incorporating benzodifurandione into the π-conjugated backbone. The introduction of an electron-deficient moiety not only results in strongly electron-deficient characteristics but also extends the conjugation and enhances the intramolecular and intermolecular interactions of the polymers. Therefore, the D-A conjugated polymer (PBIBDF-BT) had the absorption extend to the NIR region and exhibited excellent photothermal conversion efficiency (34.7%) (Yang et al., 2017). Herein, we currently introduce another isoindigo derivative (BABDF) by replacing the outer benzene rings of BIBDF with aza rings (Figure 1), an obvious next step, synthesize three BABDF-based conjugated polymers (PBABDF-BDTT, PBABDF-BT, and PBABDF-TVT) and study their photothermal properties. On the one hand, the introduction of electronegative N atom could further enhance the electron-withdrawing power of acceptor which result in strong push-pull interaction and significant red-shifts of absorption (Zhang et al., 2017a). On the other hand, the N-substitution on backbone can produce non-covalent interaction (S…N and CH….N) and endows the polymer with improved planar structure, which can also lead the redshift of absorption (Jackson et al., 2013; Zhang et al., 2019). Because of the hydrophobic conjugated polymers, it is difficult to further apply to living organisms, conjugated polymer-based NPs were prepared by using amphiphilic diblock copolymer poly(ethylene glycol)-block-poly(hexyl ethylene phosphate) (mPEG-b-PHEP) as the stabilizer using the single emulsion method. As a result, the polymer based on TVT as donor and BABDF as acceptor exhibited an excellent extinction coefficient of 55 cm^−1^·mg^−1^·mL and a high photothermal conversion efficiency of 40.7%. These demonstrate that the aza-heterocyclic BABDF unit may be promising in development of D-A conjugated polymer with high photothermal conversion efficiency.

## Experimental section

### Materials

Three conjugated polymers were synthesized. And a diblock copolymer mPEG-b-PHEP reported previously (Sun et al., 2014). bis(6-bromo-1-(4-decyltetradecyl)-2-oxo-7-azaindolin-3-ylidene)benzo[1,2-b:4,5-b′]-difuran-2,6(3H,7H)-dione (2Br-BABDF), (3,3′-didodecyl-[2,2′-bithiophene]-5,5′-diyl)bis(trimethylstannane) (2tin-BT), ((4,8-bis(5-(2-ethylhexyl)thiophen-2-yl)-6-(trimethylstannyl)benzo[1,2-b:4,5-b']dithiophen-2-yl)dimethylstannyl)methylium (2tin-BDTT), and (E)-1,2-bis(3-dodecyl-5-(trimethylstannyl)thiophen-2-yl)ethene (2tin-TVT) were synthesized according to the methods that reported in documents (Li et al., 2016b; Zhang et al., 2017a). Tris(dibenzylideneacetone)dipalladium (Pd_2_(dba)_3_), tri(o-tolyl)phosphine (P(o-tol)_3_), and other chemicals were purchased from Sigma-Aldrich Chemical Company, Alfa Aeasar Chemical Company, and Sinopharm Chemical Reagent Co. Ltd., China. Chemical regents were purchased and used as received. 3-(4,5-Dimethylthiazol-2-yl)-2,5-diphenyl tetrazolium bromide (MTT) was purchased from Sigma-Aldrich (St. Louis, MO, United States).

### Synthesis of the Polymer PBABDF-BDTT

Tris(dibenzylideneacetone)dipalladium (Pd_2_(dba)_3_, 0.004 g, 0.0044 mmol) and tri(o-tolyl)phosphine (P(o-tol)_3_, 0.0055 g, 0.018 mmol) were added to a solution of 2Br-BABDF (0.15 g, 0.11 mmol) and 2tin-BDTT (0.10 g, 0.11 mmol) in toluene (15 mL) under nitrogen. The solution was subjected to three cycles of evacuation and nitrogen filling. The mixture was then heated to 110°C for 48 h. After cooling to room temperature, the mixture was poured into methanol and stirred for 12 h. A black precipitate was collected by filtration. The product was purified by Soxhlet extractor using methanol and dichloromethane. The residue was extracted with hot chloroform in an extractor for 24 h. After removing the solvent, a black solid was collected (0.14 g, 70.5%). GPC: *Mn* = 42.5 *kDa, PDI* = 2.47. Elemental analysis: calcd for C_104_H_144_N_4_O_6_S_4_ (%): C, 74.60, H, 8.67, N, 3.35, S, 7.66. Found (%): C, 74.75, H, 8.73, N, 3.30, S, 7.58.

### Synthesis of the Polymer PBABDF-BT

The same procedure was used as PBABDF-BDTT. The compounds used were Pd_2_(dba)_3_, (0.004 g, 0.0044 mmol), P(o-tol)_3_ (0.055 g, 0.018 mmol), 2Br-BABDF (0.15 g, 0.11 mmol) and 2tin-BT (0.09 g, 0.11 mmol). After the workup, a black solid was collected (0.14 g, 72.0%). GPC: *Mn* = 40.6 *kDa, PDI* = 2.30. Elemental analysis: calcd for C_104_H_156_N_4_O_6_S_2_ (%): C, 76.99, H, 9.69, N, 3.45, S, 3.95. Found (%): C, 77.02, H, 9.87, N, 3.22, S, 3.90.

### Synthesis of the Polymer PBABDF-TVT

The same procedure was used as PBABDF-TVT. The compounds used were Pd_2_(dba)_3_, (0.004 g, 0.0044 mmol), P(o-tol)_3_ (0.055 g, 0.018 mmol), 2Br-BABDF (0.15 g, 0.11 mmol) and 2tin-TVT (0.094 g, 0.11 mmol). After the workup, a black solid was collected (0.14 g, 73.1%). GPC: *Mn* = 44.2 *kDa, PDI* = 2.12. Elemental analysis: calcd for C_106_H_158_N_4_O_6_S_2_ (%): C, 77.23, H, 9.66, N, 3.40, S, 3.89. Found (%): C, 77.70, H, 9.57, N, 3.23, S, 3.72.

### Fabrication and Characterization of Nanoparticles

A CHCl_3_ solution (200 μL) containing mPEG-b-PHEP (10.0 mg), PBABDF-BT (1.0 mg), and ultrapurified water (1.0 mL) was emulsified by ultrasound for 2 min (work 5 s and rest 2 s) at a 325 W output using a microtip probe sonicator (JY92-IIN, Scientz Biotechnology, Ningbo, China). The solution was vortexed for 30 min to remove the organic solvent and centrifuged at a speed of 3,500 for 5 min. No precipitation occurred in this way. It was then further purified by being passed through a 0.45 μm filter (Millipore). Also, the above steps were repeated s to obtain the NP_TVT_ and NP_BDTT_. The size distribution of conjugated polymer nanoparticles in aqueous solution was measured by dynamic light scattering (DLS) that conducted with a NanoBrook-90 Plus instrument (Brookhaven Instrument Corporation, Holtsville, NY, United States). The transmission electron microscope (TEM, JEOL-2010, Japan) measurements were implemented with an acceleration voltage of 200 kV.

### Measurement of Photothermal Conversion Efficiency

In order to evaluate the photothermal ability, the aqueous solution of these conjugated polymer nanoparticles (40.0 μg/mL) was placed in centrifuge tube and then illuminated with 808 nm irradiation at 2.0 W/cm^2^ (New Industries Optoelectronics, Changchun, China) for 15 min. In this process, the temperature of the solution and the infrared thermal images was recorded using an infrared camera (ICI7320, Infrared Camera Inc., Beaumont, TX, United States).

According to the reported method by Roper et al. the total energy balance for the system can be indicated by Equation 1:

(1) $$\begin{array}{r} \eta =\frac{\text{hS}\left({\text{T}}_{\text{Max}}-T_{Surr}\right)-Q_{Dis}}{\text{I}\left(1-10^{-{\text{A}}_{808}}\right)} \end{array}$$

In which, h is heat transfer coefficient, S is the surface area of the container, T_Max_ (unit: °C) and T_Surr_ (unit: °C) are the balance temperature and ambient temperature of the surroundings, respectively, Q_Dis_ is the heat induced by the light absorbance of water solvent without nanoparticle, and I (unit: mW) is the incident laser power. A_808_ is the absorbance of nanoparticle solution at 808 nm.

### Photostability

The NPs (40.0 μg/mL) were irradiated by 808 nm NIR laser (2.0 W/cm^2^, 15 min, laser on). Subsequently, the NIR laser was closed for 15 min, and then the solution was naturally cooled to room temperature. The laser on and laser off cycles were repeated three times to monitor temperature changes as described above.

### *In vitro* Cytotoxicity of NP_TVT_

Breast cancer MDA-MB-231 cells (American Type Culture Collection, Rockefeller, Maryland, United States), were seeded in a 96-well plate (1 × 10^4^ cells per well) at 37°C with 5% CO_2_ overnight. The medium was substituted by fresh medium containing NP_TVT_ at different concentrations. After incubation for 4 h, and then the cell viability was analyzed by MTT assay.

### Measurements and Characterization

Nuclear magnetic resonance (NMR) spectra were recorded on a Mercury plus 600 MHz machine. Elemental analysis was performed using a Vario EL instrument. Molecular weights were characterized by gel permeation chromatography (GPC) using a Waters Series 1525 binary HPLC pump and 1,2,4-trichlorobenznen as the eluent and polystyrene as the standard. Thermogravimetric analysis (TGA) analyses were conducted with a TA instrument QS000IR at a heating rate of 20°C min^−1^ under nitrogen gas flow. Differential scanning calorimetry (DSC) was performed on a TA instrument Q2000 under nitrogen. The sample was first cooled down to −65°C, then heated up to 250°C and held for 2 min to remove thermal history, followed by cooling at a rate of 10°C min^−1^ to −65°C and then heating at a rate of 10°C min^−1^ to 250°C in all cases. UV-vis-NIR spectra were detected using a UV-3802 (UNICO, Shanghai, China) spectrophotometer. The absorption spectra were recorded on polymer solutions in chloroform and nanoparticle solutions in water. Electrochemical measurements were conducted on a CHI 660D electrochemical analyzer under nitrogen in a deoxygenated anhydrous acetonitrile solution of tetra-n-butylammonium hexafluorophosphate (0.1 M) with a scan rate of 0.1 V/s. A platinum electrode was used as both working and auxiliary electrodes, and an Ag/Ag^+^ electrode was used as a reference electrode.

## Results and Discussion

### Synthesis and Characterization

The synthetic route of three polymers (**PBABDF**-**BDTT, PBABDF**-**BT**, and **PBABDF**-**TVT**) are shown in Figure 1. The three polymers were synthesized by Stille cross-coupling polymerization in the presence of tris(dibenzylideneacetone)dipalladium (Pd_2_(dba)_3_) as the catalyst and tri(o-tolyl)phosphine (P(o-tolyl)_3_) as the ligand. The three polymers were purified by precipitating from methanol and washing with methanol and hexane by Soxhlet extraction for 24 h to eliminate the oligomers. Then, the residue was extracted with chloroform. After concentrating the chloroform solvent under vacuum distillation, the three polymers were obtained by precipitating from methanol. The structures were characterized by nuclear magnetic resonance (NMR) (Figures S5–S7). And the molecular weights of three polymers were determined by gel permeation chromatography (GPC), using trichlorobenzene as the eluent. The number average molecular weights (M_n_) of **PBABDF**-**BDTT, PBABDF**-**BT**, and **PBABDF**-**TVT** were 42.5, 40.6, and 44.2 kDa, respectively, and the polydispersity indexes (PDI) were 2.47, 2.30, and 2.12, respectively.

As shown in Figure S1, the thermal properties of three polymers were evaluated by thermogravimetric analysis (TGA) and differential scanning calorimetry (DSC) under nitrogen atmosphere. The decomposition temperature (Td) at 5% weight loss for polymers was above 350°C, high enough for photothermal study. The DSC results did not provide any information about the glass transition temperature of polymers in the temperature range of our study.

### Optical and Electrochemical Properties

**Figure 2:**
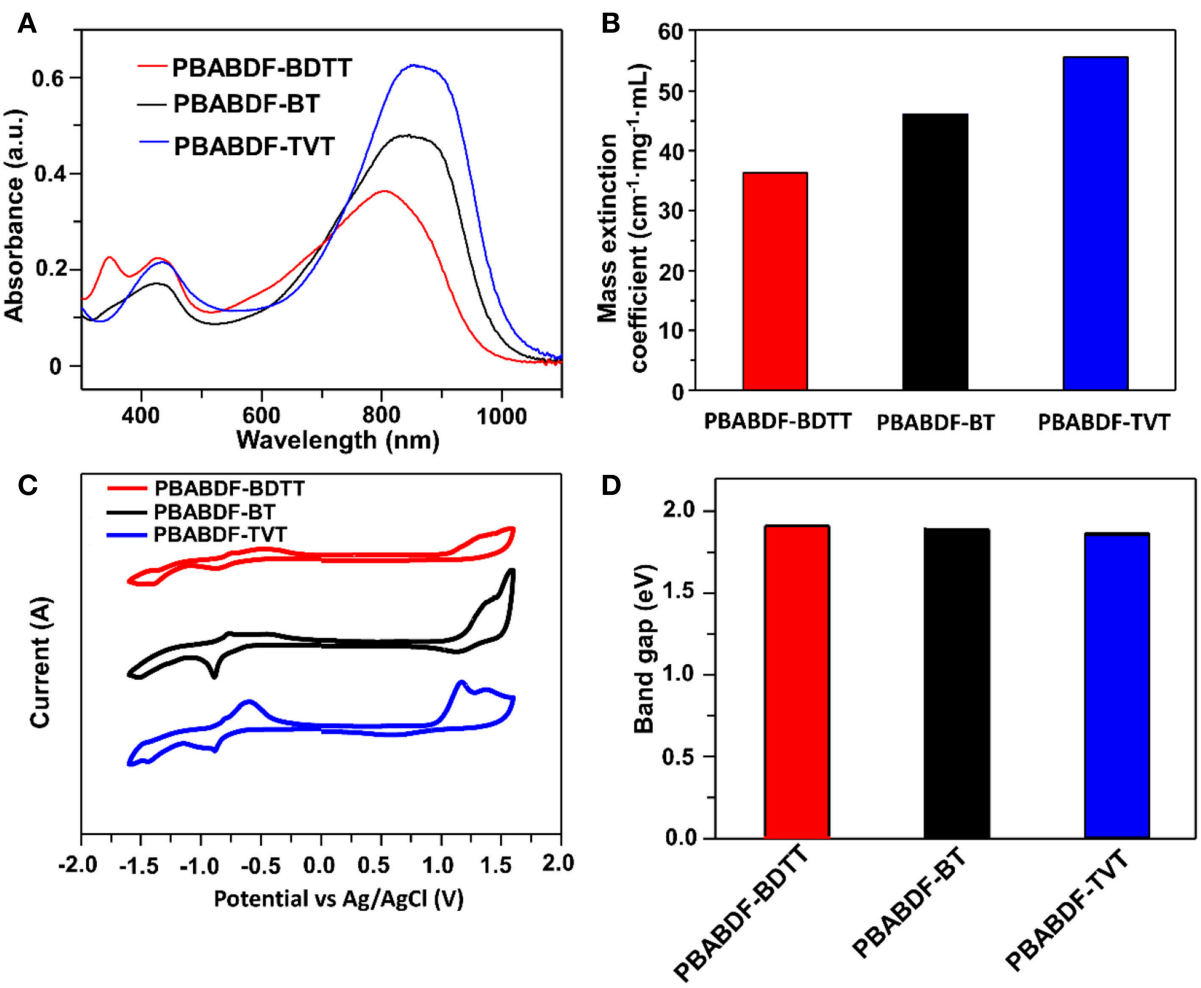
**(A)** Absorption spectra of these conjugated polymers with different D units (10.0 μg/mL). **(B)** Mass extinction coefficients at 808 nm. **(C)** Cyclic voltammetry. **(D)** The band gaps of these conjugated polymers.

The UV-vis-NIR absorption spectra of the polymers characterized in chloroform solution (10 μg/mL with 2 mL) were shown in Figure 2A. All the polymers showed typical dual-band absorption in the solution, and the absorption band-edge (λ_onset_) extend to ~1000 nm. The maximum absorption peaks were 804, 846, 853 nm for **PBABDF-BDTT, PBABDF-BT, PBABDF-TVT**, respectively. The low**-**energy band ranged from 600 nm to 1000 nm; this can be attributed to intramolecular charge transfer (ICT) from the donor to the acceptor core. The high-energy band ranged from 310 nm to 500 nm that can be assigned to the π-π^*^ transition of polymer backbone (Pierre et al., 2010; Kim et al., 2014). Moreover, the corresponding mass extinction coefficients at 808 nm (Figure 2B) were calculated to be 36.3, 46.1, and 55.7 cm^−1^·mg^−1^·mL for **PBABDF-BDTT, PBABDF-BT, PBABDF-TVT**, respectively. The absorption intensity gradually increased with increasing concentrations (Figures S2A–C), and all CPs displayed a linear relationship between absorbance and concentration at 808 nm (Figure S2D). In addition, the **PBABDF-TVT** showed a slightly red shift and strong NIR absorption compared with the other two materials. This is most probably attributed to the more planar structure of PBABDF-TVT (Lei et al., 2012). The corresponding optical bandgaps of **PBABDF-BDTT**, **PBABDF-BT**, and **PBABDF-TVT** were 1.28, 1.24, and 1.22 eV, respectively. The narrow bandgaps were due to the extended π-conjugation along the polymer backbone and may be beneficial to their photothermal conversion in NIR region (Lei et al., 2012).

The electrochemical properties of three polymers were investigated by cyclic voltammetry (CV) (Figure 2C). And the corresponding data was summarized in Table 1. Three polymers showed deep lowest unoccupied molecular orbital (LUMO) and highest occupied molecular orbital (HOMO) energy levels due to the presence of strong electron-withdrawing BABDF group. The LUMO/HOMO energy levels were −4.08/−5.80 eV, −4.11/−5.81 eV and −4.02/−5.70 eV for **PBABDF-BDTT**, **PBABDF-BT**, and **PBABDF-TVT**, respectively. As shown in Figure 2D, the bandgaps were 1.72 eV, 1.70 eV, and 1.68 eV, respectively, which are 0.4 eV higher than their optical bandgaps calculated from the absorption of their solutions. These differences may be caused by large exciton binding energy (Bredas, 2014, Zhu et al., 2016).

**Table 1:** Molecular Weight, optical and electrochemical properties of polymers.

**Polymer**	**($\lambda_{max}^{abs}$) nm**	**M_n_**	**PDI(kDa)**	**λ_onset_(nm)**	**${\text{E}}_{g}^{opt}$^a^(eV)**	**HOMO^b^ (eV)**	**LOMO^c^ (eV)**	**${\text{E}}_{g}^{ec}$^d^ (eV)**
	**solution**							
PBABDF-BDTT	804	42.5	2.47	969	1.28	−5.80	−4.08	1.72
PBABDF-BT	846	40.6	2.30	998	1.24	−5.81	−4.11	1.70
PBABDF-TVT	853	44.2	2.12	1019	1.22	−5.70	−4.02	1.68

a $E_{g}^{opt}$ =1240/λ_onset._

b HOMO = −(4.75+ $E_{onset}^{ox}$).

c LUMO = −(4.75+ $E_{onset}^{red}$).

d *$E_{g}^{ec}$ = −(HOMO–LUMO)*.

### Preparation and Characterization of NPs

**Figure 3:**
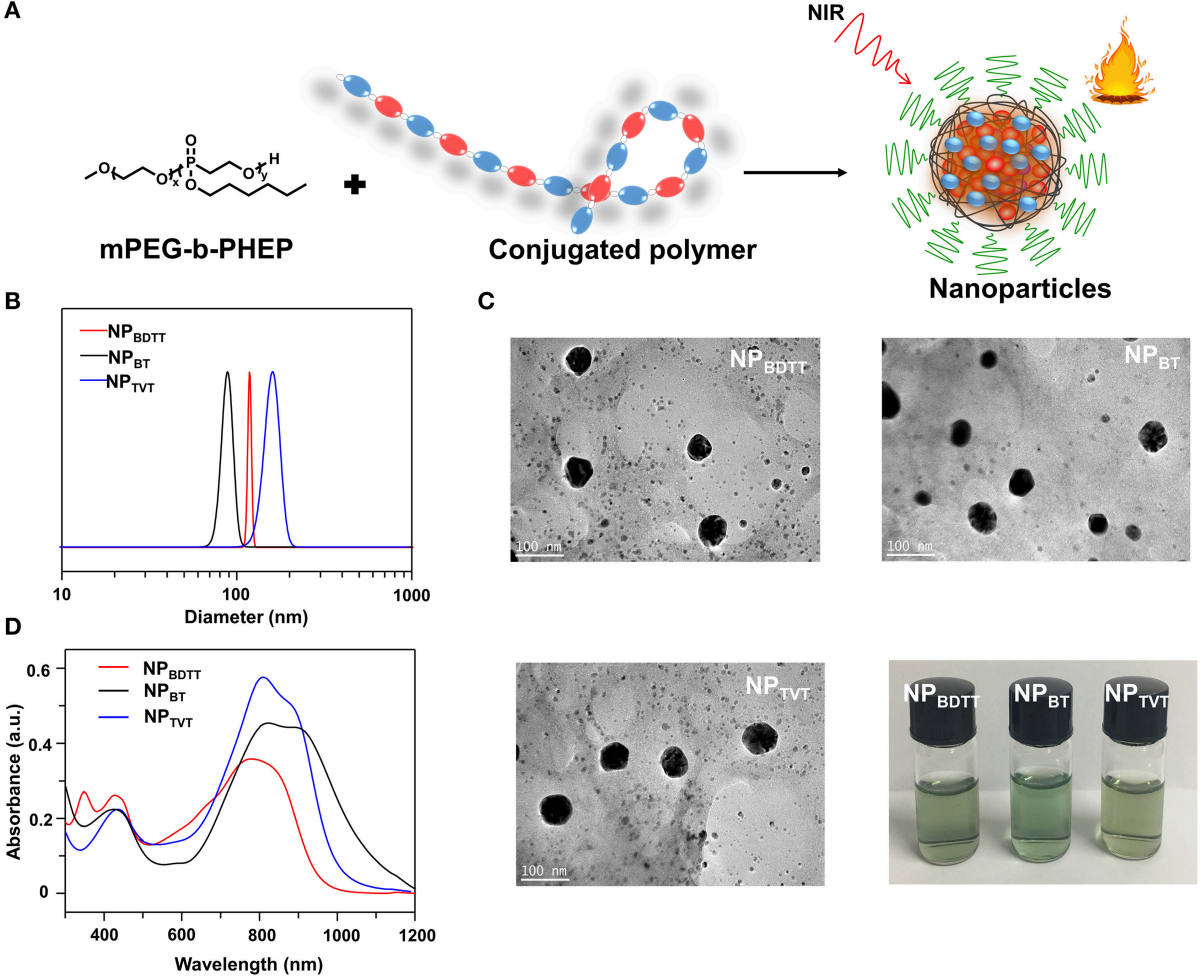
**(A)** Scheme illustration of the nanoparticles prepared. **(B)** Size distributions. **(C)** TEM images and Photographic images. **(D)** Absorption spectra of NPs (10.0 μg/mL).

To endow these polymers with good stability for subsequent applications, an amphipathic carrier mPEG-b-PHEP was used to package the D-A conjugated polymer through self-assembly and to provide a relatively inert particle surface (Figure 3A). This is the hydrophobic conjugated polymer and the hydrophobic part of the amphiphilic block copolymer gradually tangled during the evaporation of organic solvents from oil/water emulsion, which is result of the intermolecular interactions, mainly involving the hydrophilic and hydrophobic interactions. The obtained NPs were denoted as NP_BDTT_, NP_BT_, and NP_TVT_, respectively. The average size of all the obtained NPs determined by dynamic light Scattering (DLS) was about 100 nm (Figure 3B). Additionally, the transmission electron microscopy (TEM) images of NPs showed a classic spherical structure (Figure 3C). No sediment was observed for the NPs after storage at 4°C for three months (Figure 3C). These results indicated that all the NPs have good stability. The UV-vis-NIR absorption spectra of the polymer nanoparticles in water obtained at the same concentration (10 μg/mL with 2 mL) were shown in Figure 3D. NP_BDTT_, NP_BT_, and NP_TVT_ exhibited absorption peaks at 799 nm, 821 nm and 819 nm, respectively, which are blue-shifted by about 5 nm, 25 nm and 32 nm compared to the absorption peaks from these conjugated polymers. Moreover, the corresponding mass extinction coefficients of NPs at 808 nm were calculated to be 35.1, 45.2, and 57.5 cm^−1^·mg^−1^·mL, respectively. The results showed that the extinction coefficient of NPs was similar to those of conjugated polymers. The corresponding data was summarized in Table 2.

**Table 2:** Optical and fluorescence quantum yield of NPs.

**Nanoparticle**	**$\lambda_{max}^{abs}$(nm)**	**ε ^808^ (cm^−1^·mg^−1^·mL)**	**Quantum yield, QY(%)**
	**Solution**		
NP_BDTT_	799	35.1	0.012
NP_BT_	821	45.2	0.015
NP_TVT_	819	57.2	0.010

### Photothermal Performance of NPs

**Figure 4:**
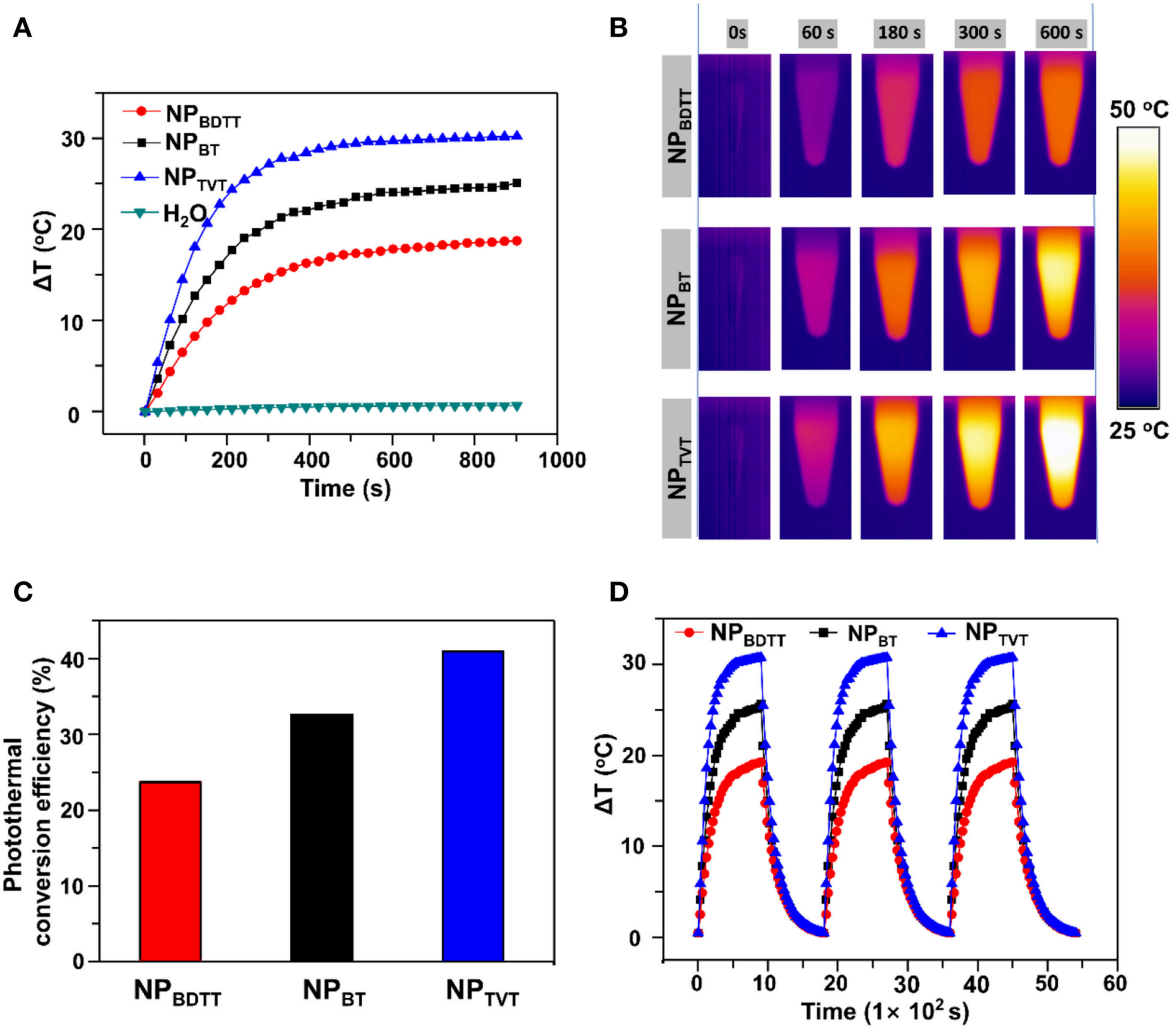
**(A)** Temperature change curves of the nanoparticles upon exposure to NIR laser (808 nm, 2.0 W/cm^2^, 15 min). **(B)** Thermal images of NPs upon exposure to the NIR laser (808 nm, 2.0 W/cm^2^). **(C)** Photothermal conversion efficiency. **(D)** Temperature elevation of NPs (40.0 μg/mL) over three laser on/off cycles of NIR irradiation (808 nm, 2.0 W/cm^2^, 15 min).

To demonstrate the photothermal conversion ability of NPs, the temperature change was monitored under 808-nm irradiation at 2.0 W/cm^2^. NPs with a concentration of 40.0 μg/mL were irradiated for 15 min, the infrared thermal images of nanoparticle aqueous solution were recorded. As the irradiation time increased, the temperature of particle solution gradually increased in the following order: NP_BDTT_ < NP_BT_ < NP_TVT_ (Figure 4A). After the irradiation for 15 min, the temperature changes of NP_BDTT_, NP_BT_, and NP_TVT_ increased by 18.78, 25.16, and 30.27°C, respectively. In addition, the temperature of all the NPs monotonically increased with increasing concentration, and the temperature increased of NPs at each concentration followed this order (Figure S3). The infrared imaging color also showed the temperature difference between these NPs. As shown in Figure 4B, the color of photothermal images changed from violet (low temperature) to bright yellow (high temperature). According to the method developed by Roper et al. (2007), the photothermal conversion efficiencies (η value) of NPs were calculated (see calculation details in Supporting Information). As shown in Figure 4C and Figure S4, the η values of NP_BDTT_, NP_BT_, and NP_TVT_ were calculated to be 23.5, 32.4, and 40.7%, respectively. Further, the photostability of these NPs was studied. Three cycles of laser on/off (time: 15 min) with NIR irradiation (808 nm, 2.0 W/cm^2^) were performed. Compared to the temperature change after the first laser irradiation, the temperature showed no significant reduction for all these NPs for another two cycles (Figure 4D). Moreover, the NPs were exposed to the 808-nm NIR laser of 2.0 W/cm^2^ for 15 min until steady-state temperature was reached. Figure 4D showed that all the NPs reached a certain temperature and remained unchanged, indicating that they had excellent NIR photostability. The excellent photostability of NPs can be attributed to the thermal properties of semiconducting polymers. As mentioned before, their decomposition temperatures were above 350°C (Figure S1), which was enough for the photothermal effect.

### Interpretation of Photothermal Performance

**Figure 5:**
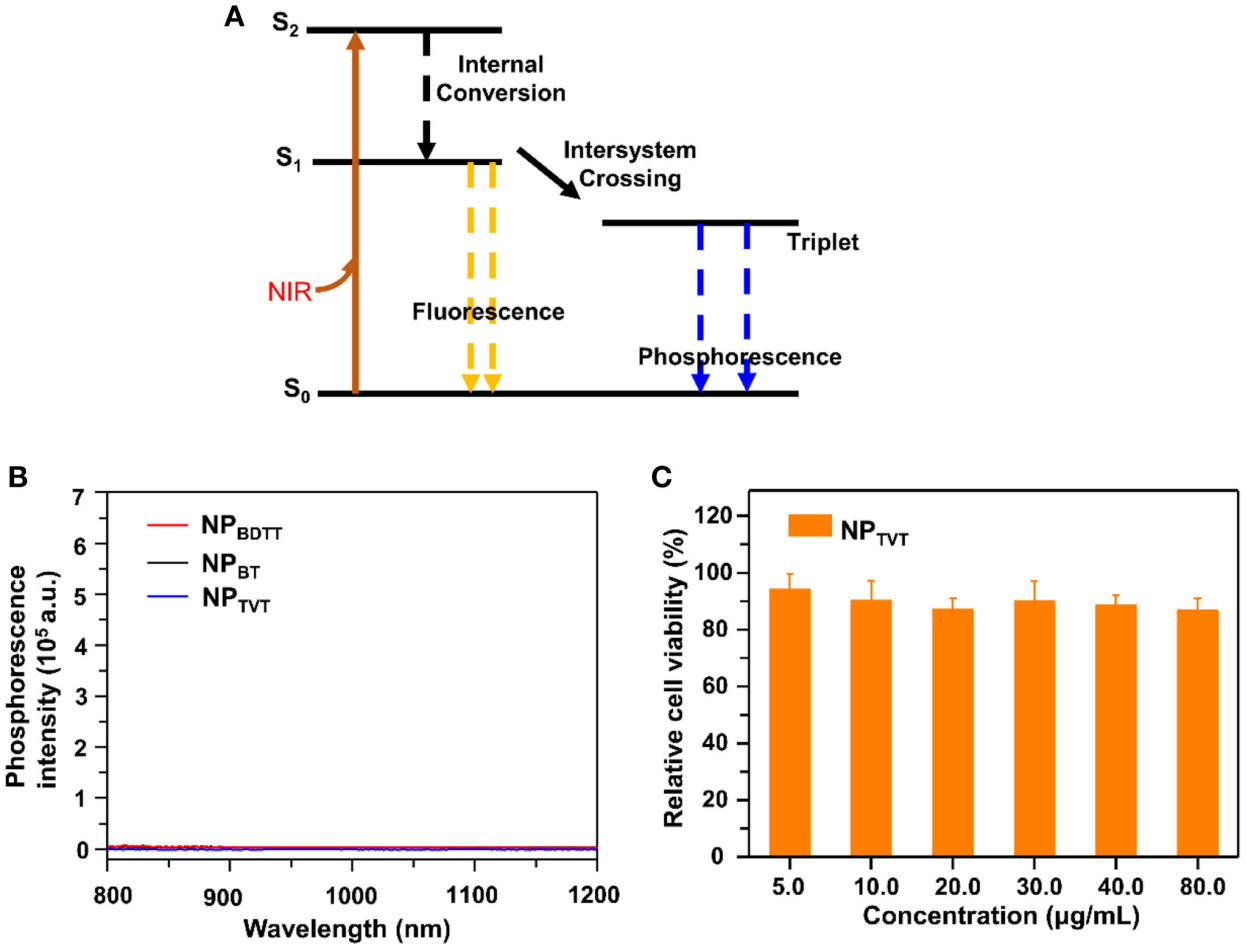
**(A)** Energy level diagram. **(B)** Phosphorescence spectra of NP_BDTT_, NP_BT_, NP_TVT_ upon excitation at 808 nm. **(C)** Cell viability of 231 cells in the presence of NPs treated with different concentrations.

The abovementioned results indicated that the photothermal effect of NPs decreased in the following order: NP_TVT_ > NP_BT_ > NP_BDTT_. When nanoparticle solutions are irradiated by a beam of light, it produces a range of possible physical phenomena, such as scattering (including the result of multiple refractions and reflections), absorption, and luminescence. We set the intensity of the incident light to I_incident_, the intensity of the scattered light to I_scat_, and the intensity of the absorbed light to I_abs_, then I_incident_ = I_scat_ + I_abs_, and the absorbance of light can be calculated as: Q_abs_ = I_abs_**/**I_incident_. As shown in Figure 5A, the absorbed light energy usually undergoes (i) fluorescence emission, (ii) non-radiation (thermal energy loss), and (iii) intersystem conversion to long-lived species (e.g., phosphorescence) (Silvia and George, 1992). If nanoparticle solutions are not chemiluminescent or negligible, then the absorbed light energy could be eventually converted into almost all heat. To prove the photothermal effect of these NPs in such an order, the photothermal conversion efficiency, absorption spectrum, fluorescence quantum yield, phosphorescence, and mass extinction coefficient were tested. All NPs showed almost no fluorescence emission (Table 2). And phosphorescence spectra of NPs upon excitation at 808 nm were shown in Figure 5B. It can be seen that their absorption was negligible. Therefore, the generation of heat may be the main way to eliminate the absorption of energy after NIR irradiation. The mass extinction coefficient of NPs was calculated from its absorption spectrum, which are 35.1, 45.2, and 57.5 cm^−1^·mg^−1^·mL, respectively. NP_TVT_ absorbed more light energy at the same concentration compared to NP_BDTT_. Therefore, the photothermal performance of NP_TVT_ was significantly higher than that of NP_BDTT_. These results showed that photothermal effect was related to the mass extinction coefficient. The strongest absorbance at 808 nm ensured the highest photothermal effect of NP_TVT_, followed by NP_BT_ and NP_BDTT_.

### *In vitro* Cytotoxicity of NP_TVT_

To achieve the full potential of NP_TVT_ in biomedical fields, 3-(4,5-Dimethylthiazol-2-yl)-2,5-diphenyl tetrazolium bromide (MTT) assay was used to detect the cytotoxicity of NP_TVT_. MDA-MB-231 cells were selected as cancer cell model. MDA-MB-231 cells were incubated with NP_TVT_ at different PBABDF-TVT concentrations. As shown in Figure 5C, with the increase in concentration, no obvious cytotoxicity and proliferation inhibition of 231 cells were observed. Notably, incubation with NP_TVT_ nanoparticles, even at the highest concentration, also did not exhibit obvious toxicity to MDA-MB-231 cells without NIR light illumination. This indicated that the NP_TVT_ nanoparticle is biocompatible, and because of its high photothermal conversion efficiency, it can be further applied to organisms for cancer photothermal therapy.

## Conclusion

Here, a series of polymers with different chemical structures were synthesized by varying the donor moieties. A systematic study showed that the chemical structure of a strong acceptor and suitable donor unit has a significant effect on the absorption spectrum, extinction coefficient, and photothermal conversion efficiency of polymers. This study demonstrated that high extinction coefficient, excellent photothermal conversion efficiency, good biocompatibility, and high stability of conjugated polymers can be obtained through rational molecular design by introducing a strong electron-withdrawing electron acceptor.

## Data Availability

All datasets generated for this study are included in the manuscript and/or the Supplementary Files.

## Author Contributions

GZ, LQ, and YD conceived and designed the experiments. LQ supervised the project. SM performed the experiments. JR, LT, HL, WW, and YZ assisted in the preparation of the manuscript. GZ and SM wrote the paper. All authors analyzed the data.

### Conflict of Interest Statement

The authors declare that the research was conducted in the absence of any commercial or financial relationships that could be construed as a potential conflict of interest.
