# Supplementary material for: Aza-Based Donor-Acceptor Conjugated Polymer Nanoparticles for Near-Infrared Modulated Photothermal Conversion
Source: Front Chem. 2019 May 21;7:359. doi: 10.3389/fchem.2019.00359 (PMC6536684; doi:10.3389/fchem.2019.00359)
Supplement: Supplementary file 1 [file Data_Sheet_1.docx]

Supplementary Material

**The calculation of photothermal conversion efficiency**

According to the reported method by Roper et al, the total energy balance for the system can be expressed by Eq. 1:

$\text{ƞ=}\frac{\text{hS}\left( \text{T}_{\text{Max}}\text{-}\text{T}_{\text{Surr}} \right)\text{-}\text{Q}_{\text{Dis}}}{\text{I(1-}\text{10}^{\text{-A}_{\text{808}}}\text{)}}$ **(1)**

Take NP_TVT_ as an example, where *T_Max_* (unit: °C) and *T_Surr_* (unit: °C) are the balance temperature and ambient temperature of the surroundings, respectively. (*T_Max_ - T_Surr_*) was 30.27 °C according to Figure S4b. *Q_Dis_* is the heat induced by the light absorbance of water solvent without NP_TVT_ and it was measured to be 1.9 mW. I (unit: mW) is the incident laser power (600 mW). A_808_ is the absorbance (1.78) of NP_TVT_ at 808 nm. Thus, only the *hS* remains unknown for calculating. h is heat transfer coefficient, S is the surface area of the container.

In order to get the unknown parameter *hS*, a dimensionless driving force temperature, is introduced by using the maximum system temperature, noted as *Tmax*

$\text{Ɵ=}\frac{\text{T-}\text{T}_{\text{Surr}}}{\text{T}_{\text{Max}}\text{-}\text{T}_{\text{Surr}}}$ **(2)**

At the cooling stage of aqueous dispersion of the NPTVT, the light source was shut off, and the value of $\text{τ}$_s_ (155.06 s) is obtained (Figure S4a) according this Eq. 3.

$\text{T=-}\text{τ}_{\text{S}}\text{lnθ}$ **(3)**

and the value of *hS* is derived according to Eq. 4.

$\text{τ}_{\text{S}}\text{=}\frac{\sum\text{m}_{\text{i}}\text{C}_{\text{p,i}}}{\text{hS}}$ **(4)**

Where the *C* is 4.2 J**·**g^-1^**·**°C^-1^ and the *m* is 0.3 g. Therefore, following the Eq. 4, the *hS* is calculated to be 8.13 mW/°C. Substituting the value of *hS* into Eq. 1, the 808 nm laser heat conversion efficiency (ƞ) of NP_TVT_ can be calculated to be 40.7%. Similarly, other nanoparticles ƞ was calculated such as the above statement.

**
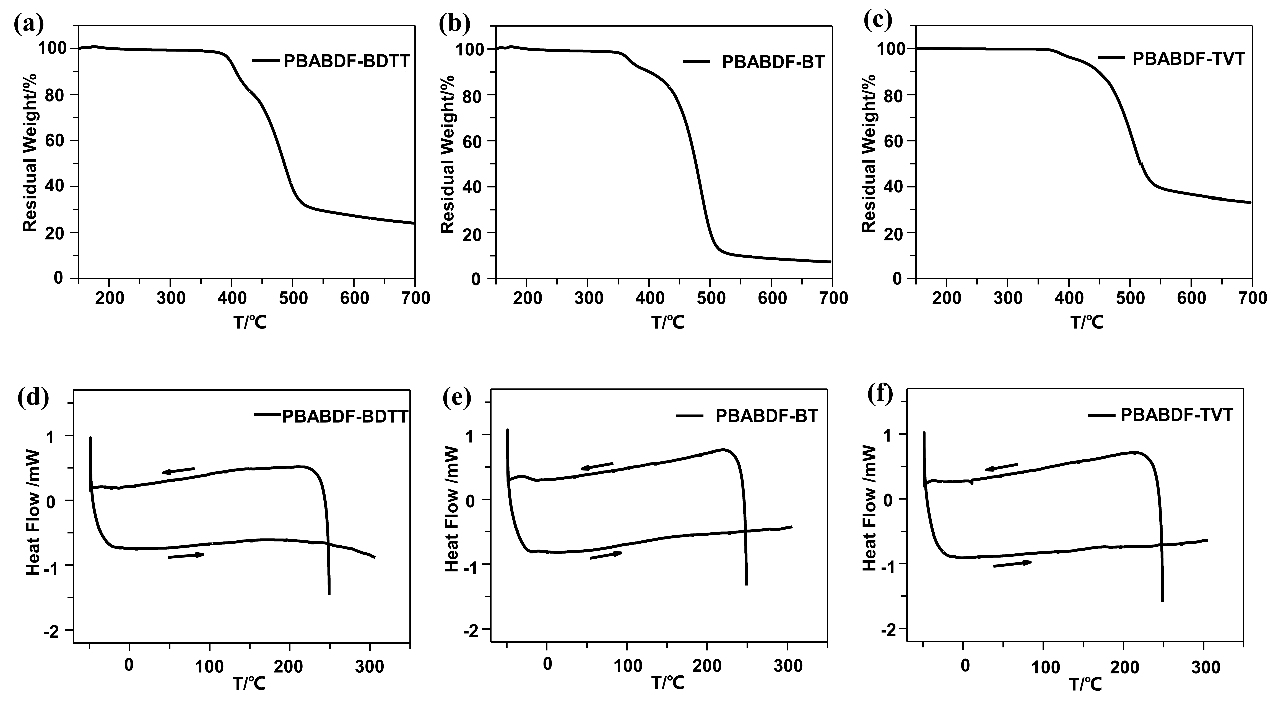
**

Figure S1. TGA (a), (b), (c) and DSC (d), (e), (f) analysis of these conjugated polymers.


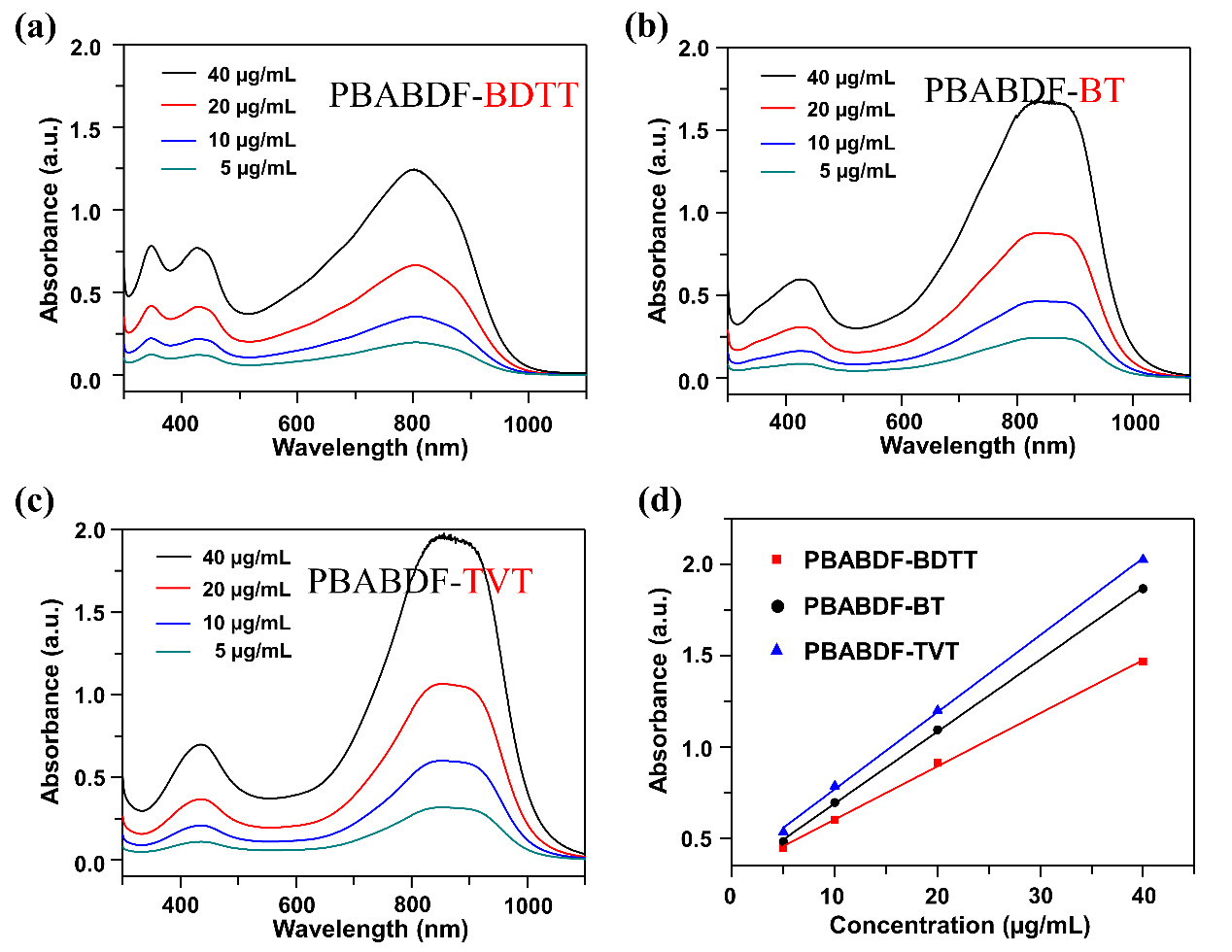


Figure S2. (a), (b), (c) The concentration independence of polymers. (d) Linear fitting plots of absorbance and the conjugated polymers concentration.


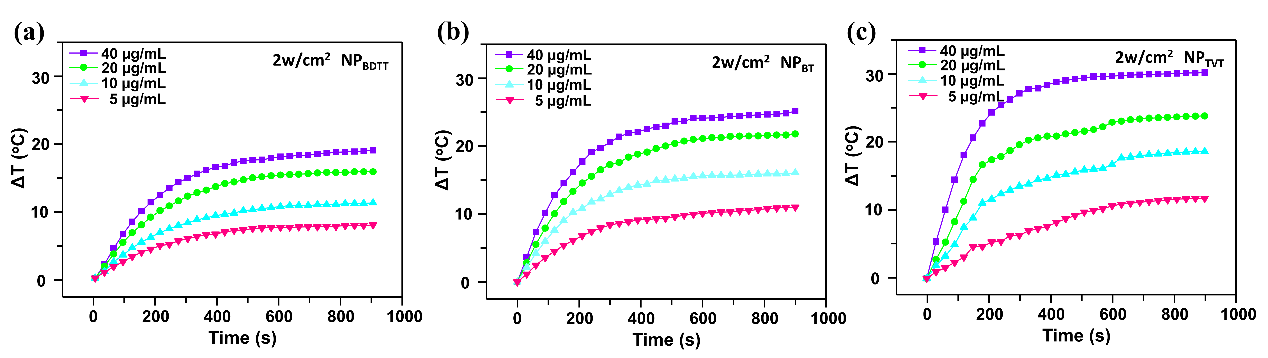


Figure S3. Temperature change curves of at different concentrations upon exposure to the NIR laser (808 nm, 2.0 W/cm^2^, 15 min).


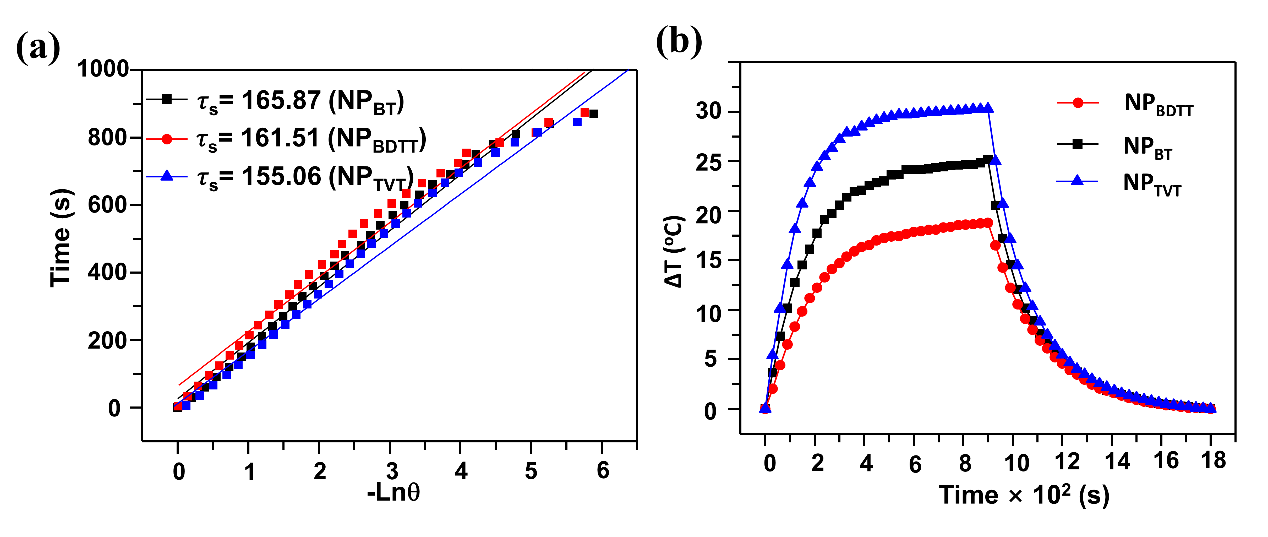


Figure S4. (a) Time constant for heat transfer from the system is determined to be τ_s_ by applying the linear time data from the cooling period versus negative natural logarithm of driving force temperature, which is obtained from the cooling stage of panel A. (b) Photothermal effect of the aqueous dispersion of these nanoparticles (40 μg/mL) irradiated with 808 laser (a power density of 2.0 W/cm^2^), in which the irradiation lasted for 15 min, and then the laser was shut off.

**
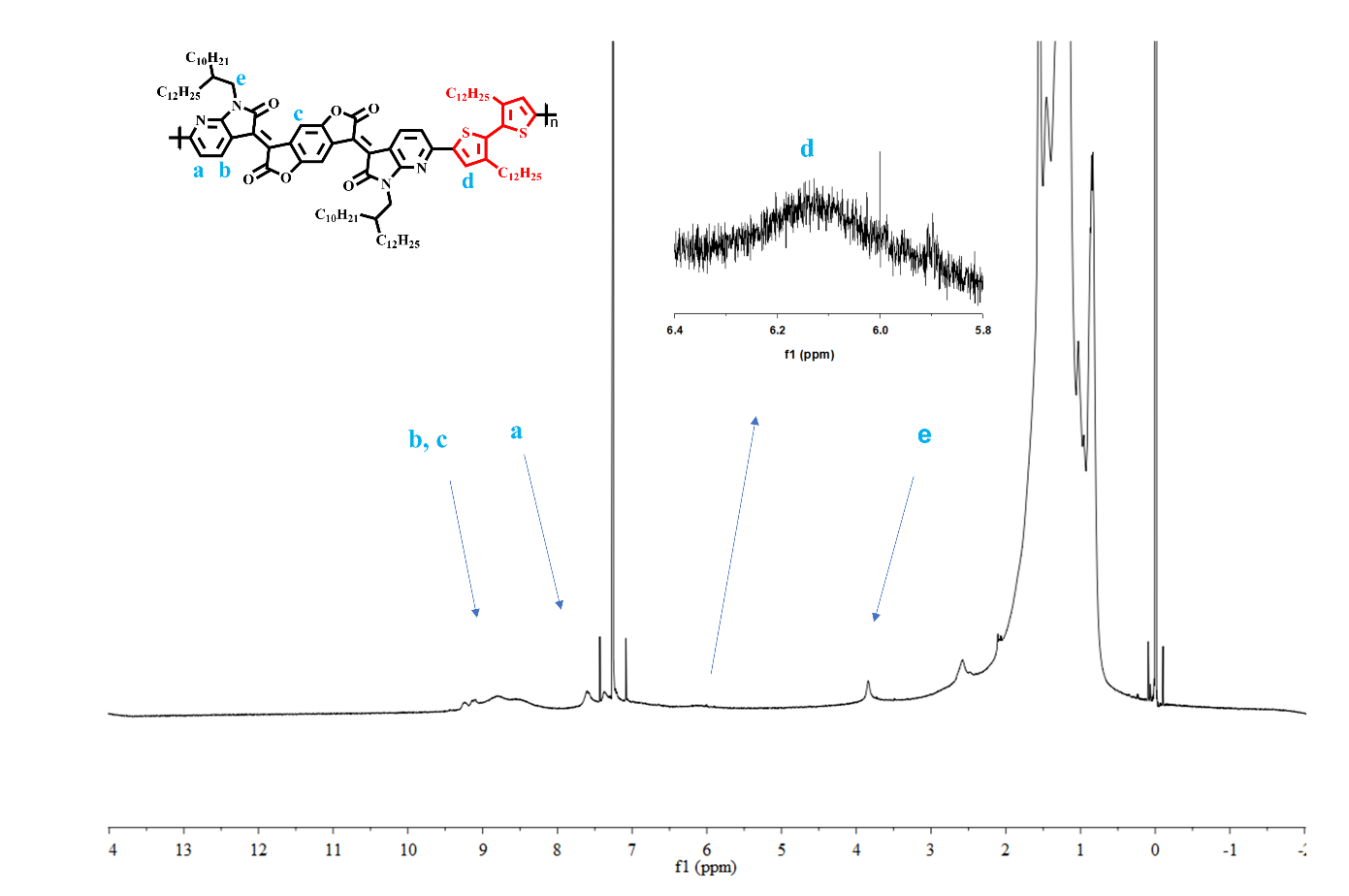
**

Figure S5. ^1^H NMR spectrum of PBABDF-BT in CDCl_3_.

**
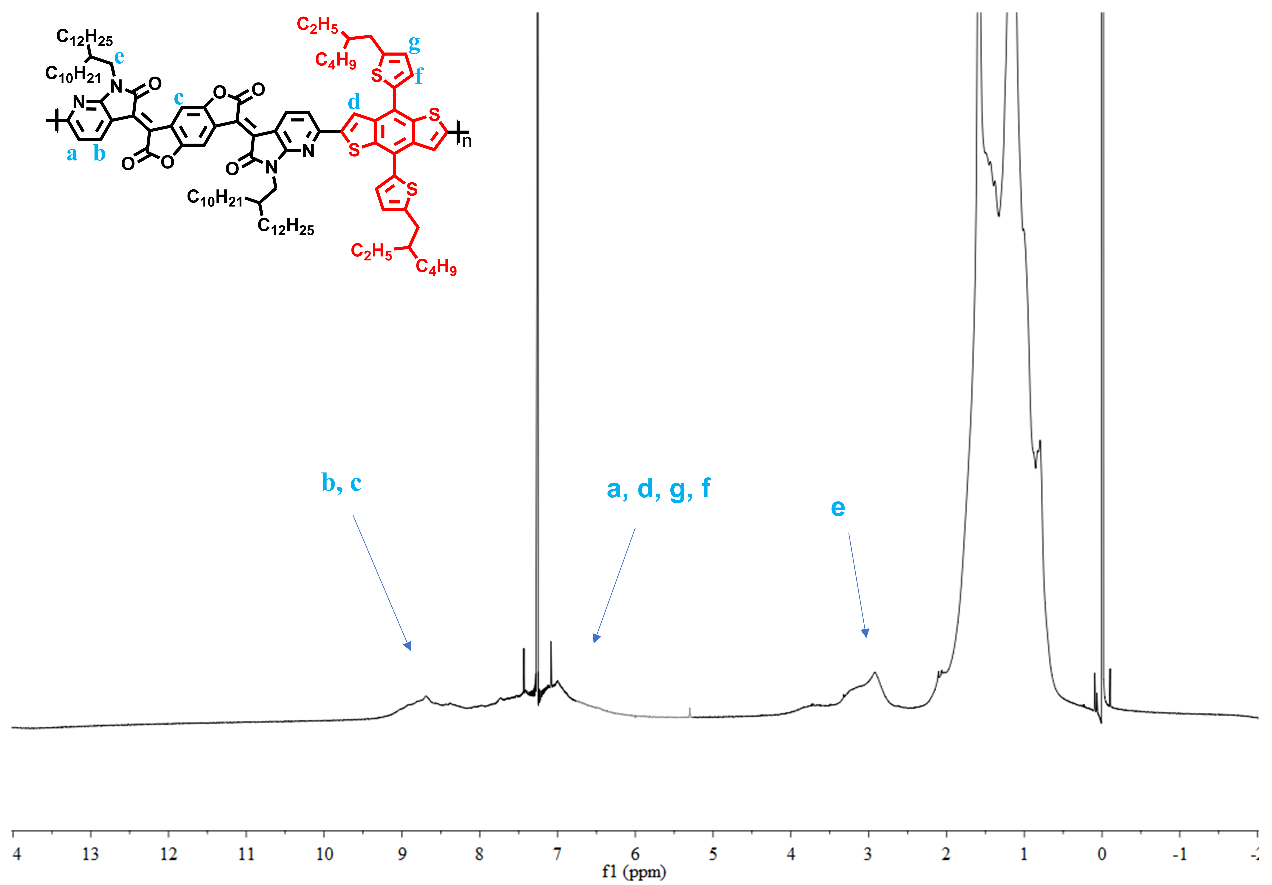
**

Figure S6. ^1^H NMR spectrum of PBABDF-BDTT in CDCl_3_.

**
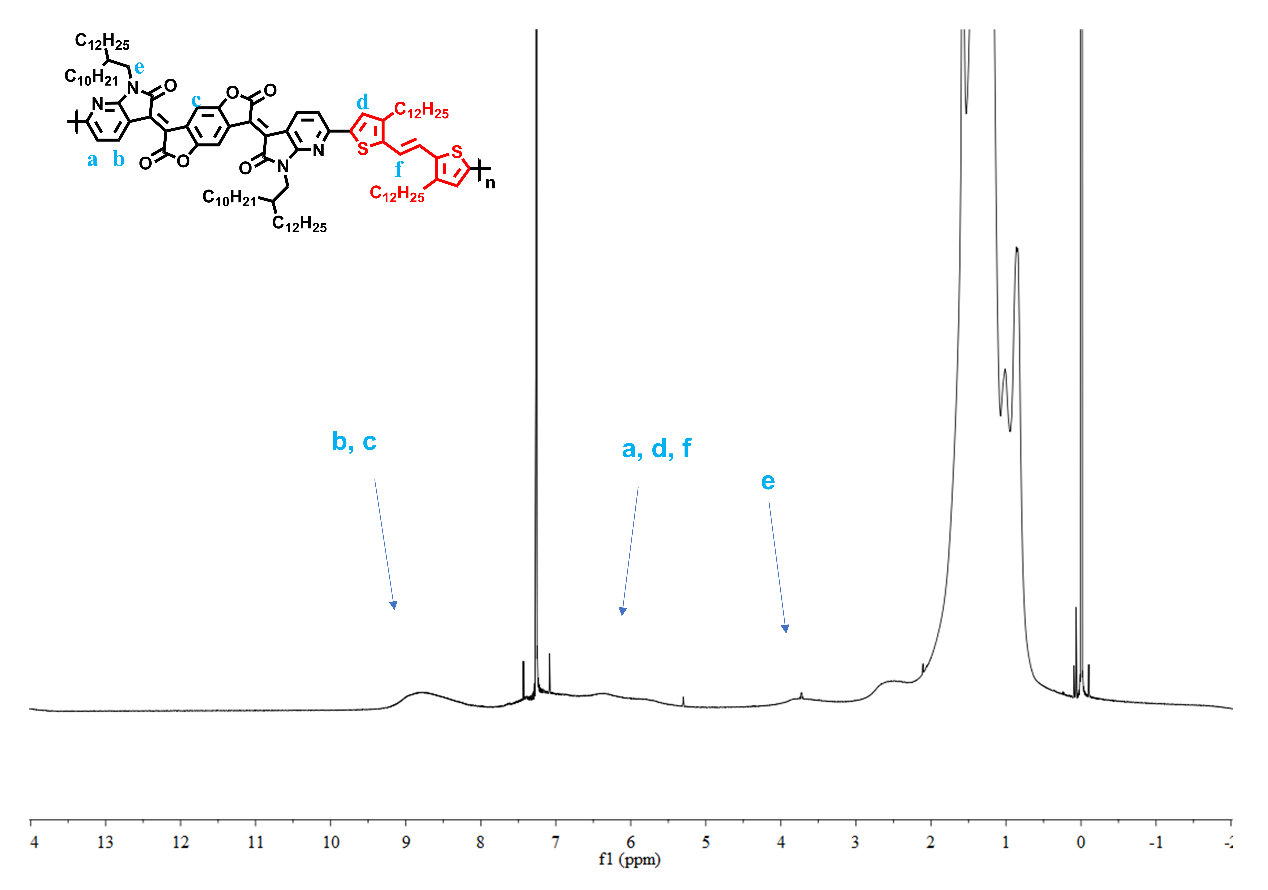
**

Figure S7. ^1^H NMR spectrum of PBABDF-TVT in CDCl_3_.
